# Editorial: Addiction and the brain: current knowledge, methods, and perspectives

**DOI:** 10.3389/fpsyt.2023.1343524

**Published:** 2023-12-20

**Authors:** Hao Chen, Sören Kuitunen-Paul, Aviv M. Weinstein, Johannes Petzold

**Affiliations:** ^1^Department of Psychiatry and Psychotherapy, Technische Universität Dresden, Dresden, Germany; ^2^Department of Clinical Psychology and Psychotherapy, Technische Universität Chemnitz, Chemnitz, Germany; ^3^Department of Child and Adolescent Psychiatry, Technische Universität Dresden, Dresden, Germany; ^4^Department of Psychology, Ariel University, Ariel, Israel

**Keywords:** addiction, substance use disorder (SUD), non-invasive brain stimulation, relapse prediction, addiction treatment, behavioral addictions

Addiction is commonly considered a disorder that affects the brain and changes behavior. Substance use disorders, among the leading causes of death and disability (1), continue to be major public health challenges. Behavioral addictions, which share certain neurobiological mechanisms with substance use disorders (2), have received increasing attention over the last two decades. Yet, we lack an overarching theoretical framework that integrates the advancements in neurobiological research with the development, progression, and treatment of addiction.

Despite the significant progress in our understanding of addiction (3–5), the translation of this knowledge into effective treatment options remains a critical challenge (6). In this Research Topic, we present selected studies that aim to bridge this gap by carefully assessing relevant cohorts, by evaluating available brain-related interventions, or by developing innovative approaches to the treatment of substance use disorders (see Table 1 for overview).

**Table 1:** Overview of edited primary studies on addictions.

**Authors**	**Keyword(s)**	**Substance, diagnosis**	**Setting of treatment/recruitment**	**Country**	Sample size, female %^*^	**Participant age range (mean)**	**Study type**	**Intervention/treatment (experimental)**	**Additional naturalistic treatment**	**Primary outcome variable(s)**	**Follow-up interval**
**Studies on brain-related intervention effects**											
Chen J. et al.	Resting-state connectivity	Heroin/methadone, HUD	Heroin treatment program including methadone maintenance treatment	China, East Central	*N* = 94 (*N* = 37 HUD, *N* = 57 controls); 10% and 8% female	— (*M* = 37 and *M* = 35)	Observational, longitudinal, self-controlled, quasi-experimental	—	Methadone maintenance treatment including monthly random urine testing	Coupling of default mode and salience networks, changes in psychological characteristics	One year (HUD group only)
Rosenthal et al.	Meditation	Alcohol, AUD	*Ad-hoc* community sample^**^	Europe, Germany	*N* = 62 (*N* = 27 AUD, *N* = 35 controls); 17% and 59% female	— (*M* = 39 and *M* = 38)	Randomized, within-subject	Audio-guided body scan meditation against a control condition (audio of nature sounds)	—	Pavlovian-to-instrumental transfer effect	Within treatment session
van Oort et al.	Resting-state connectivity	Alcohol, AUD	Inpatient AUD treatment center with detoxification	USA, northeast	*N* = 64 (*N* = 37 inpatients, *N* = 27 controls); 40% female	30–59 years (*M* = 47 and *M* = 47)	Prospective, quasi-experimental, randomized, naturalistic	—	NIAAA treatment program for AUD, including group and individual therapy and pharmacological interventions when appropriate	Left and right frontoparietal networks connectivity, default mode network connectivity	Treatment entry (baseline) to treatment end (follow-up) = 4 weeks ± 9 days
Gullett et al.	Resting-state connectivity	Alcohol	*Ad-hoc* community sample	USA, southeast	*N* = 35 with heavy alcohol use; 40% female	45–75 years (*M* = 57)	Prospective, one-group, controlled, within-subject	Contingency management aiming at drinking reduction rather than abstinence	—	Resting-state functional connectivity of the salience network	30 days
**Studies on brain-centered interventions**											
Hu et al.	rTMS	Alcohol, AUD	Inpatient and outpatient treatment centers (different hospitals)	China (multiple)	*N* = 263; 3.0%−15.2% female	— (*M* = 44–48)	Prospective, randomized, double-blind, sham-controlled	Ten sessions rTMS at DLPFC across 2 weeks (starting at baseline) plus either (a) 8 × 60 min CBT across 8 weeks (starting at baseline) or (b) 1 × 10 min clinical interview	Mecobalamin, vitamin B, vitamin C, vitamin E. Temporary short-term low-dose benzodiazepines when appropriate	Relapse (combining self-reports and family member telephone interviews)	6 months following discharge
Upton et al.	rTMS (cTBS, iTBS)	Nicotine, ND	*Ad-hoc* community sample	USA, midwest	*N* = 31; 48% female	— (*M* = 47)	Prospective, within-subject	Two randomized, counterbalanced, neuronavigated TBS sessions to the rIFG—one administering cTBS, and the other administering iTBS	—	Smoking behaviors, fronto-striatal-limbic resting-state functional connectivity	Within treatment session
Dong et al.	rTMS (iTBS)	Heroin & methamphetamine concurrently, HUD & MUD	Inpatient addiction treatment center	China, East Central	*N* = 56; 16% female	40–62 years (—)	Prospective	Twenty sessions of rTMS to the DLPFC	Unspecified inpatient treatment as usual including pharmacological interventions when appropriate	Cognitive functioning, 10 related protein markers in blood serum	Treatment entry (baseline) to treatment end (follow-up) = 4 weeks
Chen Y.-H. et al.	rTMS, tDCS	Methamphetamine, MUD	Clinical (review)	—	—	—	Review	rTMS, tDCS, (EEG-fNIRS for assessment)	—	—	—
**Studies on relapse prediction using brain parameters**											
Sasaki et al.	fNIRS	Alcohol, AUD	Inpatient treatment centers	East Asia, Japan	*N* = 41; 14.6% female	— (*M* = 51.6–55.0)	Prospective, controlled	—	Detoxification treatment (1–2 weeks, including diazepam infusions), subsequent inpatient treatment (3.5 months, treatment based on “12 Step” meetings), optional post-discharge services (outpatient visits, daycare activities, self-help groups)	Associations between relapse status and possible predictors measured during hospitalization (notably task-related brain treatment measured via fNIRS)	6 months following discharge
Martelli et al.	Structural MRI	Alcohol, AUD	Inpatient treatment centers	Europe, France	*N* = 23 (*N* = 17 inpatients, *N* = 6 healthy controls); no females	— (*M* = 50.8–54.9)	Prospective, controlled	—	Detoxification treatment finished	Association between AUD/relapse status and regional cerebral volumes	7 years
**Studies on comorbidities with a possible shared brain mechanism**											
Shen et al.	Oxytocin receptor polymorphism	Alcohol, AUD	Hospitals with inpatient detoxification treatment	China, North	*N* = 265; no females	— (*M* = 45)	Non-interventional, cross-sectional	—	Detoxification treatment finished	Interactions between polymorphism and self-reported anxiety & depression	—
Luderer et al.	Comorbidity	Alcohol, AUD	Inpatient and outpatient psychiatric treatment institution	Europe, Germany	*N* = 47 patients (*N* = 6 AUD only, *N* = 12 AUD + ADHD, *N* = 19 ADHD only); 6% and 50% and 68% female	— (*M* = 44 and *M* = 39 and *M* = 30)	Non-interventional, cross-sectional	—	—	Comparison of diagnostic utility between self-report scale and a continuous performance test	—
Miller et al.	Cohort	Gambling, GD	Outpatient treatment center	Europe, Sweden	*N* = 204; 26.4% female	— (*M* = 36.1)	Non-interventional, cross-sectional, cohort	—	CBT	Demographics, GD severity, prevalence of other psychiatric diagnoses, additional addictive behaviors, quality of life, gambling-related cognitive distortions	—

* Recalculated for this table when only group sample sizes were presented in the respective paper.

** Including persons with AUD diagnosis but no necessity for detoxification.

—, not reported or not applicable.

AUD, alcohol use disorder; CBT, cognitive-behavioral therapy; cTBS, continuous theta-burst stimulation, a patterned form of rTMS; DLPFC, dorsolateral prefrontal cortex; EEG, electroencephalography; fNIRS, functional near-infrared spectroscopy; GD, gambling disorder; HUD, heroin use disorder; iTBS, intermittent theta-burst stimulation, a patterned form of rTMS; MUD, methamphetamine use disorder; MRI, magnetic resonance imaging; NIAAA, National Institute on Alcohol Abuse and Alcoholism in the USA; ND, nicotine dependence; tDCS, Transcranial direct-current stimulation; rIFG, right inferior frontal gyrus; rTMS, repetitive transcranial magnet stimulation.

## Studies on brain-related intervention effects

Chen J. et al. evaluated the commonly adopted treatment approach, methadone maintenance treatment, for heroin use disorder, within a 1-year longitudinal study. The results confirmed the effectiveness of methadone in reducing withdrawal symptoms and preventing relapses. At the imaging level, increased connectivity within the default mode network (DMN) was associated with reduced withdrawal symptoms, while the increased connectivity between the DMN and the salience network might pose risks of relapse given its link to enhanced salience signal of heroin cues. Clinicians may need to evaluate both positive and negative effects of this treatment approach during application.

Mindfulness-based interventions, rooted in neurobiological findings and increasingly being adopted in treatment centers globally, have also emerged as a powerful treatment approach for substance misuse (7), offering the added advantages of ease of access and low costs. Rosenthal et al. aimed to better understand the underlying mechanisms of a short, guided meditation by assessing how changes in environmental cues influence instrumental behaviors in a Pavlovian-to-instrumental transfer (PIT) task. The meditation reduced the PIT effect in individuals with alcohol use disorder (AUD), but not in the control group. This pilot study paves the way for future research to further assess the effectiveness of mindfulness-based interventions and to better understand their cognitive mechanisms.

Another promising approach for the development of personalized treatments and recovery is to address problems in early abstinence and their underlying mechanisms. van Oort et al. studied brain network connectivity to find such mechanisms, which may ultimately help individuals to better maintain abstinence. In a related study, Gullett et al. investigated participants (heavy alcohol use; with or without HIV) who attempted abstinence for 30 days via contingency management. Lower baseline connectivity in the salience network, which is linked to susceptibility to environmental cues, predicted reduction in drinking. Although this finding highlights a promising target for intervention, individuals living with HIV, who tend to have lower baseline connectivity in the salience network, may not benefit as much from contingency management as those without HIV.

## Studies on brain-centered interventions

Three studies evaluated non-invasive brain stimulation for treatment, highlighting it as a promising tool owing to its safety, precision, and importantly, potential for combination with other treatments. Hu et al. demonstrated the effectiveness of reducing relapse rates by combining repetitive transcranial magnetic stimulation (rTMS) and cognitive behavioral therapy in a clinical trial with 263 participants diagnosed with alcohol dependence. Building on the concept of rTMS, theta burst stimulation (TBS)—including continuous TBS (cTBS) and intermittent TBS (iTBS)—represents another innovative approach while being safe and efficacious (8). Upton et al. demonstrated the benefits of cTBS on the right inferior frontal gyrus in reducing cravings for smoking and increasing resting-state fronto-striatal functional connectivity over 24 h in individuals with nicotine dependence. Dong et al. investigated patients with polydrug (heroin and methamphetamine) use disorder and revealed the superior effect of iTBS compared to rTMS and sham iTBS in improving cognitive functions, thus highlighting its clinical value.

In their review, Chen Y.-H. et al. propose an intelligent closed-loop TMS neuromodulation system that is informed and repeatedly adapted via measurements from multimodal electroencephalogram–functional near-infrared spectroscopy (EEG-fNIRS) in order to treat methamphetamine addiction and methamphetamine-related craving. This innovative approach has the potential to improve clinical outcomes by providing real-time monitoring and intervention for patients seeking to achieve abstinence from drug use.

All these findings collectively underscore the promise and potential of non-invasive brain stimulation techniques, such as rTMS and TBS, in offering new and effective treatment modalities for various forms of addiction.

## Studies on relapse prediction using brain parameters

While non-invasive brain stimulation has shown promising results, it is important to comprehend the mechanisms that cause some individuals to maintain abstinence while others relapse post-treatment. Two studies aimed to identify (bio)markers predictive of future relapses in individuals with AUD. Sasaki et al. measured fNIRS during cognitive tasks and identified reduced brain responses in right frontotemporal areas to emotional stimuli, along with risk-seeking behavior, as markers for relapse within 6 months. In a 7-year follow-up study, Martelli et al. identified a larger caudate volume as a biomarker for relapse. These studies highlight the potential for identifying specific biomarkers that can predict relapse, thus providing a valuable direction for future research and more individualized interventions.

## Studies on comorbidities with a possible shared brain mechanism

Complementing the two studies that identified specific biomarkers predictive of relapse, Shen et al. provided further insight into the genetic factors that may influence withdrawal symptoms in individuals with AUD. The identification of the oxytocin receptor rs2254298 polymorphism as a significant modulator of mood disorders during alcohol withdrawal adds to our understanding of the genetic basis of addiction and withdrawal. This finding highlights the importance of personalized treatments that consider both genetic and environmental factors.

Given AUD often co-occurs with other mental disorders (9), Luderer et al. investigated the relationship between attention-deficit/hyperactivity disorder (ADHD) and AUD across many dimensions. Hyperactivity emerged as a significant symptom in individuals with both ADHD and AUD, indicating a treatment target for individuals with both conditions.

Lastly, Miller et al. addressed gender differences in gambling disorder, which is particularly relevant given its escalating prevalence and the notable overrepresentation of affected men (10). The study underscored the distinct motivations, patterns, and consequences of gambling behavior between men and women, thereby paving the way for more targeted and effective interventions. This may, in the future, include non-invasive rTMS given that neurobiological links have been found between gambling disorder and several of the substance-related use disorders (11) for which rTMS has been shown to be promising by authors in this Research Topic (Chen Y.-H. et al.; Dong et al.; Hu et al.; Upton et al.).

## Conclusion

The studies presented in this Research Topic provide exciting insights into the current developments in neurobiologically informed addiction treatment, from traditional to innovative techniques. Several of the presented findings highlight the potential for new and effective treatment modalities that consider the neurobiological mechanisms underlying addiction, as well as the need for personalized interventions informed by both genetic and environmental factors. As we continue to explore the complexities of addiction, it is our hope that these insights will help develop more effective and targeted treatments, ultimately improving outcomes for individuals struggling with substance use disorders and behavioral addictions.

## Author contributions

HC: Writing—original draft. SK-P: Validation, Visualization, Writing—review & editing. AW: Writing—review & editing. JP: Validation, Writing—review & editing.

## References

[1] Castelpietra G Knudsen AKS Agardh EE Armocida B Beghi M Iburg KM. The burden of mental disorders, substance use disorders and self-harm among young people in Europe, 1990–2019: findings from the Global Burden of Disease Study 2019. Lancet Reg Health Eur. (2022) 16:100341. 10.1016/j.lanepe.2022.100341 35392452 PMC8980870

[2] Yau YHC Leeman RF Potenza MN. Biological underpinning of behavioral addictions and management implications. In el-Guebaly N, Carrà G, Galanter M, Baldacchino AM, editors. Textbook of Addiction Treatment: International Perspectives. Berlin: Springer International Publishing (2021), p. 889–910. 10.1007/978-3-030-36391-8_63

[3] Heilig M MacKillop J Martinez D Rehm J Leggio L Vanderschuren LJMJ. Addiction as a brain disease revised: why it still matters, and the need for consilience. Neuropsychopharmacology. (2021) 46:10. 10.1038/s41386-020-00950-y 33619327 PMC8357831

[4] Volkow ND Koob GF McLellan AT. Neurobiologic advances from the brain disease model of addiction. N Engl J Med. (2016) 374:363–71. 10.1056/NEJMra1511480 26816013 PMC6135257

[5] Wiers RW Verschure P. Curing the broken brain model of addiction: neurorehabilitation from a systems perspective. Addict Behav. (2021) 112:106602. 10.1016/j.addbeh.2020.106602 32889442

[6] Verdejo-Garcia A Lorenzetti V Manning V Piercy H Bruno R Hester R. A roadmap for integrating neuroscience into addiction treatment: a consensus of the neuroscience interest group of the international society of addiction medicine. Front Psychiatry. (2019) 10:877. 10.3389/fpsyt.2019.00877 31920740 PMC6935942

[7] Li W Howard MO Garland EL McGovern P Lazar M. Mindfulness treatment for substance misuse: a systematic review and meta-analysis. J Subst Abuse Treat. (2017) 75:62–96. 10.1016/j.jsat.2017.01.008 28153483

[8] Oberman L Edwards D Eldaief M Pascual-Leone A. Safety of theta burst transcranial magnetic stimulation: a systematic review of the literature. J Clin Neurophysiol. (2011) 28:67. 10.1097/WNP.0b013e318205135f 21221011 PMC3260517

[9] Kessler RC Crum RM Warner LA Nelson CB Schulenberg J Anthony JC. Lifetime co-occurrence of DSM-III-R alcohol abuse and dependence with other psychiatric disorders in the National Comorbidity Survey. Arch Gen Psychiatry. (1997) 54:313–21. 10.1001/archpsyc.1997.01830160031005 9107147

[10] Potenza MN Balodis IM Derevensky J Grant JE Petry NM Verdejo-Garcia A. Gambling disorder. Nat Rev Dis Primers. (2019) 5:1. 10.1038/s41572-019-0099-7 31346179

[11] Grant JE. Neurobiology of disordered gambling. Curr. Addict. Rep. (2016) 3:445–9. 10.1007/s40429-016-0119-6

